# Everybody's Page

**Published:** 1892-07-09

**Authors:** 


### Everybody's Page.

Strikes have recently shown a new development. In Spain the Stock Exchange for a few hours joined the merry band of protesting idlers, and the costermongers of Madrid showed their sympathy with the wrongs of their fellow men on 'Change by following their example, which was kind and considerate of the costermongers.

Whereas the English language is rapidly covering the face of the world, English weights and measures are rapidly retiring from the stage. They will no longer appear in the United States Pharmacopœia, their place being taken in the new edition, which is in course of preparation, by the metric system.

It will be interesting to see the reasons given for the several answers to the truly awful conundrum set as a prize question by *Woman*. The question is a truly feminine one, and, like many a feminine query, is a poser. It is, "Would you rather marry a man whom you entirely love, but whose love for you you are not sure of; or a man who entirely loves you, but whose love you do not feel able thoroughly to reciprocate?"

Those who have fallen victims to the reprehensible practice of packing jam in glass jars will be glad to hear that the jams manufactured by Hodson, of North Tadworth, will in future be packed only in earthenware and stone jars. This system should undoubtedly be adopted by all factories, for numerous people have met with serious injuries from time to time inflicted by pieces of glass in jam which has been supplied in glass jars.

It is unfortunate that the efforts of the Government in India to destroy poisonous snakes should be checkmated to a great extent by the attitude taken by the people towards these undesirable reptiles. Only low caste natives will endeavour to exterminate them, owing to the fact that in many districts snakes are objects of veneration and worship. Apart from this, Government measures might, perhaps, be carried further, and in addition to the reward given for every dead snake brought to the civil authority of the district, a reward, as suggested by Surgeon-general Francis, might be with advantage given for every snake's egg taken.

Opposition seems to be one of the surest and speediest ways to promote the ultimate success of most causes, however hopeless this attainment may appear at the outset. How true this is is exemplified by the position held in different countries in the world of medicine by homœopathy. The strides made by this school of medicine in Europe have never been great, though little or no obstacles have been placed in it way. In America its position is different. An exaggerated opposition has had the inevitable result of gaining some ardent champions on its behalf, and a sympathy ever readily afforded, rightly or wrongly, to a downtrodden cause, which has enabled homœopathy to take its place on a firm footing as a distinct school of medicine.

It is asserted that mosquitos can be persuaded to play the rôle of the parting guest by evaporating gum camphor in the rooms to which they have not been welcomed. The process can be accomplished in a very simple manner by placing a piece of the gum camphor in a plate, and holding it over a flame until the atmosphere is charged with the fumes.

Doctors are sometimes puzzled by the want of effect they reasonably expected from the administration of certain drugs. Can this occasionally be due to the fact that the drugs used are not pure? It is idle to suppose that pharmacy stands apart from every other trade, and is wholly free from the taint of adulteration and dishonesty. Therefore, it behoves people to show due care in having their prescriptions made up, and not to sacrifice everything to cheapness, which can be quite as great a curse as it can be a blessing.

The nature of that remarkable quack remedy "Vitaline," which was used a short while ago on a large scale in Russia, has at last been disclosed. Its popularity was naturally great, seeing that it was claimed for it that it restored to the aged their youth and long-lost vigour. The discovery of its nature was brought about by the death of a patient who had submitted to an injection; but surely the death was not caused by the use of borax and glycerine, the sole components of this marvellous "cure." Unfortunately, all quack remedies are not so simple in their nature as "Vitaline."

The economic doctrine that you should sell in the dearest market and buy in the cheapest has commended itself at least to the majority of every class in this country, and the establishment of enormous stores for the sale of goods of every kind at low prices, is one of the logical outcomes of the adoption of this doctrine. But it may be asked whether a good deal is not sacrificed sometimes by the anxiety to buy in the cheapest market. Unfortunately quality has not always been maintained with a reduction of price, which has been undoubtedly accompanied in many cases by adulteration. Cheap drugs are, therefore, not always a thing wholly to be desired, and in some cases may in the long run prove very expensive.

Attention has been drawn recently in our columns to the system of colonies for epileptics which have met with such pronounced success in Germany and America. The question very naturally arises, why cannot we do likewise? There is no doubt as to our conservatism which is often mistimed, and yearly we seem to be getting slower and slower in the adoption of new ideas. Time was when England set the example in most things to the rest of the world, whereas now it takes a deal of pushing to make us follow examples, the value of which we do not attempt to deny. Our moral digestion seems as slow as our physical. Two things are essential to the happiness of the lives of epileptics, fresh air and unobtrusive sympathy; both of which are given to them in the colonies, which might and no doubt will, at our leisure find their counterpart in this country.

